# Low LINC00599 expression is a poor prognostic factor in glioma

**DOI:** 10.1042/BSR20190232

**Published:** 2019-04-02

**Authors:** Qiang Fu, Shaoshan Li, Qingjiu Zhou, Kugeluke Yalikun, Dilimulati Yisireyili, Ming Xia

**Affiliations:** Department of Neurosurgery, The First Affiliated Hospital of Xinjiang Medical University, No.1 Liyushan Road, Urumqi 830054, Xinjiang, China

**Keywords:** biomarkers, cancer, giloma, large intervening non-coding RNA, LINC00599, prognosis

## Abstract

LINC00599 has been suggested to be involved in physiological and pathological processes including carcinogenesis. However, the clinical and prognostic significance of LINC00599 in glioma patients and the effect of LINC00599 on glioma cell migration and invasion remain unknown. In our results, we first observe the expression of LINC00599 in 31 types of human cancers including tumor tissues and corresponding normal tissues at The Cancer Genome Atlas (TCGA) database, and found that LINC00599 expression levels were only reduced in lower grade glioma (LGG) tissues and glioblastoma multiforme (GBM) tissues compared with normal brain tissues. Moreover, we confirmed levels of LINC00599 expression were decreased in glioma tissues and cell lines compared with matched adjacent normal tissues and normal human astrocytes (NHAs), respectively. Meanwhile, we found that glioma tissues with WHO III-IV grade exhibited lower levels of LINC00599 expression than glioma tissues with I-II grade. The survival analysis at TCGA data showed low LINC00599 expression was associated with poor disease-free survival and overall survival in glioma patients. *In vitro* study suggested up-regulation of LINC00599 depressed glioma cell migration and invasion through regulating epithelial–mesenchymal transition (EMT) process. In conclusion, LINC00599 acts as a tumor-suppressing long non-coding RNA (lncRNA) in glioma.

## Introduction

Glioma is the most common malignancy of the central nervous system accounting for approximately 296851 newly diagnosed cases worldwide in 2018 [2]. Meanwhile, glioma accounts for 2.5% of all cancer deaths (approximately 241037) worldwide in 2018 [2]. In the past decades, there were great improvements in treatment strategies (surgery, radiotherapy, chemotherapy, and immunotherapy) for glioma patients, but the glioma patient’s clinical outcome remained unfavorable, especially in glioblastoma paitents [1,20,24]. Hence, it is essential to elucidate mechanisms underlying glioma tumorigenesis and identify more precise prognostic biomarkers for developing effective therapeutic targets and improving clinical outcome(s).

Long non-coding RNAs (lncRNAs) are a class of transcripts that are greater than 200 nts in length and lack of coding potential [11,13]. Nowadays, more and more lncRNAs have been suggested to be dysregulated in glioma tissues and involved in regulating glioma cell proliferation, differentiation, apoptosis, cell cycle, and autophagy [21–23,27]. LINC00599 (also known as retinal non-coding RNA3) is an lncRNA transcribed from the intergenic regions of the genome and is conserved in mammals [5,25]. Originally, LINC00599 was found to be dynamically expressed and acts as a potential regulator of neurons and oligodendrocyte differentiation during retinal development [12]. In addition, abnormalities in central nervous system were observed in LINC00599^−/−^ mice, such as small brain size, axonal mis-sprouting of dentate gyrus granule cells and retinal cone cell death [15]. Subsequently, LINC00599 was shown to be involved in atherosclerosis-related vascular dysfunction and diabetes mellitus-1 related retinal microvascular dysfunction [16,17]. Recently, the function of LINC00599 in human cancer is gradually attracting the attention of scientists. In prostate cancer, LINC00599 functioned as a tumor-promoting lncRNA in regulating prostate cancer cell proliferation and invasion, and predicted tumor progression and poor survival of prostate cancer patients [19]. In glioblastoma, LINC00599 was showed to be down-regulated in tumor cell lines, and function as a tumor-suppressive lncRNA in regulating glioma cell proliferation and apoptosis [26]. However, the clinical and prognostic significance of LINC00599 in glioma patients and the effect of LINC00599 on glioma cell migration and invasion were still unclear. Therefore, we estimated the correlation between LINC00599 expression and clinicopathological characteristics in glioma patients. Moreover, we conducted gain-of-function study to investigate the impact of LINC00599 on glioma cell migration and invasion.

## Materials and methods

### Ethics statement

The study involved with human tissue specimens was approved by the Ethics Committee of The First Affiliated Hospital of Xinjiang Medical University. All participators signed the informed consent and were aware of the study detail.

### Clinical tissue specimens

A total of 60 pairs of fresh tumor tissues and matched adjacent normal tissues were collected from glioma patients who have had surgical resection at The First Affiliated Hospital of Xinjiang Medical University. All tissue specimens were pathologically confirmed by at least two pathologists, and preserved in −80°C until RNA analysis. None of glioma patients received preoperative antitumor therapy.

### The Cancer Genome Atlas database

Analysis of The Cancer Genome Atlas (TCGA) database was performed at the GEPIA (Gene Expression Profiling Interactive Analysis) platform. The difference of LINC00599 expression was observed in 31 types of human cancers including tumor tissues and corresponding normal tissues. TCGA GBM (glioblastoma multiforme, *n*=161) and LGG (lower grade glioma, *n*=514) cohorts were applied to survival analysis based on LINC00152 expression levels.

### Cell lines

Normal human astrocytes (NHA) and four human glioma cell lines (SHG-44, SW1783, U251, and LN229) were maintained with Dulbecco’s modified Eagle’s medium (DMEM; Gibco, Grand Island, NY, U.S.A.) containing 10% fetal bovine serum (FBS; Gibco, Grand Island, NY, U.S.A.) in a humidified incubator at 37°C with 5% CO_2_.

### RNA isolation and quantitative real-time PCR

Total RNAs were extracted from clinical tissue samples and cell lines using TRIzol reagent (Invitrogen, Carlsbad, CA, U.S.A.), and was reverse-transcribed into cDNA with a random primer and reverse transcriptase kit (Takara, Dalian, China) according to manufacturer’s instructions. Then, quantitative real-time PCR (qRT-PCR) was performed using TB Green Premix ExTaq II (Takara, Dalian, China) at an Applied Biosystems 7500 Real Time PCR system (Applied Biosystems, Foster City, CA, U.S.A.) based on the manufacturers protocols. The specific primers for LINC00599 were (Forward) 5′-CAACACCTTCCTCCGTGACTGTG-3′ and (Reverse) 5′-GCTGGCTCCTTCTTGTCCACATA-3′. The specific primers for GAPDH were (Forward) 5′-CGCTGAGTACGTCGTGGAGT-3′ and (Reverse) 5′-CGTCAAAGGTGGAGGAGTGG-3′. Relative LINC00599 expression was normalized to GAPDH.

### Cell transfection

The full length of LINC00599 was amplified and inserted into pcDNA3.1 plasmid (Invitrogen, Carlsbad, CA, U.S.A.) as LINC00599 overexpression vectors (pcDNA-LINC00599). Nonspecific sequence was inserted into pcDNA3.1 plasmid as negative control vectors (pcDNA-NC). The transient transfection for pcDNA-LINC00599 or pcDNA-NC plasmid in glioma cells was conducted using Lipofectamine 3000 Transfection Reagent (Invitrogen, Carlsbad, CA, U.S.A.) according to the manufacturer’s protocols.

### Transwell cell migration and invasion assays

The transwell chambers with 8‐μm pore size polycarbonate membranes (BD Biosciences, San Jose, CA, U.S.A.) were used for cell migration assay, and Matrigel–coated transwell chambers were used for cell invasion assay. Briefly, transfected glioma cells were added to the upper chambers in serum-free medium, and medium with 15% FBS was added to the lower chambers as enticement. After 24 h incubation, glioma cells at the upper surface of membranes were removed by a cotton swab, and glioma cells under glioma were fixed with methanol and stained using 0.1% Crystal Violet. The number of cells was calculated in five random fields per well under the microscope.

### Western blot

The glioma cells were harvested and lysed with radioimmunoprecipitation assay (RIPA) lysis buffer (Beyotime, Beijing, China), and the protein concentration was measured with BCA Protein Assay kit (CWBio, Jiangsu, China). Equal amount of proteins were separated in 10% SDS/polyacrylamide gel, and then transferred on to a polyvinylidene difluoride membrane (Millipore, Billerica, MA, U.S.A.). After blocking in 5% nonfat milk for 2 h at room temperature, membranes were incubated with primary antibodies: anti-E-cadherin, anti-Vimentin, or anti-β-actin (1:1000 dilution; Cell Signaling Technology, Beverly, MA, U.S.A.) at 4°C overnight. After washing, membranes were incubated with horseradish peroxidase–conjugated secondary antibodies for 2 h at temperature. Then, immunoblots were visualized by enhanced chemiluminescence (CWBio, Jiangsu, China). Finally, Quantity One Software (Bio-Rad, Hercules, CA, U.S.A.) was used for analyzing the relative integrated density values. β-actin served as the control.

### Statistical analysis

All statistical analyses were performed by SPSS 22.0 statistical software (IBM Corp., Armonk, NY, U.S.A.). Student’s *t* test was used to evaluate significant differences between independent two groups. Survival curves were plotted using the Kaplan–Meier method and compared by log-rank test. A *P*-value <0.05 was considered statistically significant.

## Results

### LINC00599 expression is reduced in glioma tissues and cell lines

**Figure 1:**
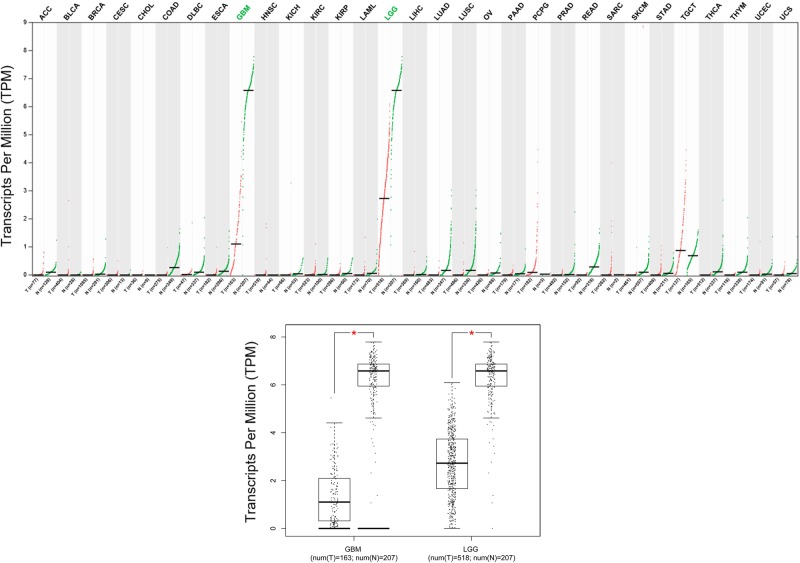
The expression pattern of LINC00599 in human cancers The expression of LINC00599 was shown in 31 types of human cancers including tumor tissues and corresponding normal tissues (ACC; BLCA; BRCA; CESC; CHOL; COAD; DLBC; ESCA; GBM; HNSC; KICH; KIRC; KIRP; LAML; LGG; LIHC; LUAD; LUSC; OV; PAAD; PCPG; PRAD; READ; SARC; SKCM; STAD; TGCT; THCA; THYM; UCEC; UCS; *, *P*<0.001).

**Figure 2:**
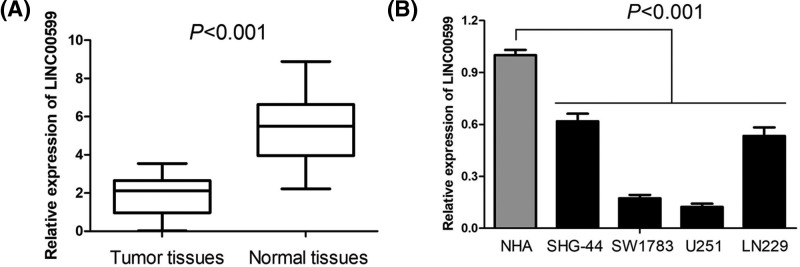
The expression pattern of LINC00599 in glioma tissues and cell lines (**A**) LINC00599 expression was remarkably decreased in glioma tissues compared with matched adjacent normal tissues. (**B**) Levels of LINC00599 expression in four human glioma cell lines (SHG-44, SW1783, U251, and LN229) were lower than that in NHA.

For exploring the expression pattern of LINC00599 in glioma, we first observe the expression of LINC00599 in 31 types of human cancers including tumor tissues and corresponding normal tissues (ACC, Adrenocortical carcinoma; BLCA, Bladder Urothelial Carcinoma; BRCA, Breast invasive carcinoma; CESC, Cervical squamous cell carcinoma and endocervical adenocarcinoma; CHOL, Cholangio carcinoma; COAD, Colon adenocarcinoma; DLBC, Lymphoid Neoplasm Diffuse Large B-cell Lymphoma; ESCA, Esophageal carcinoma; GBM; HNSC, Head and Neck squamous cell carcinoma; KICH, Kidney Chromophobe; KIRC, Kidney renal clear cell carcinoma; KIRP, Kidney renal papillary cell carcinoma; LAML, Acute Myeloid Leukemia; LGG; LIHC, Liver hepatocellular carcinoma; LUAD, Lung adenocarcinoma; LUSC, Lung squamous cell carcinoma; OV, Ovarian serous cystadenocarcinoma; PAAD, Pancreatic adenocarcinoma; PCPG, Pheochromocytoma and Paraganglioma; PRAD, Prostate adenocarcinoma; READ, Rectum adenocarcinoma; SARC, Sarcoma; SKCM, Skin Cutaneous Melanoma; STAD, Stomach adenocarcinoma; TGCT, Testicular Germ Cell Tumors; THCA, Thyroid carcinoma; THYM, Thymoma; UCEC, Uterine Corpus Endometrial Carcinoma; UCS, Uterine Carcinosarcoma), and found the expression of LINC00599 was only reduced in LGG tissues and GBM tissues compared with normal brain tissues (both *P*<0.001, Figure 1). Furthermore, we confirm the expression of LINC00599 in glioma tissues and cell lines through qRT-PCR. As shown in the Figure 2A, LINC00599 expression was remarkably decreased in glioma tissues compared with matched adjacent normal tissues (*P*<0.001). Moreover, levels of LINC00599 expression in four human glioma cell lines (SHG-44, SW1783, U251, and LN229) were lower than that in NHA (*P*<0.001, Figure 2B).

### Low LINC00599 expression is associated with high WHO grade and poor prognosis

**Figure 3:**
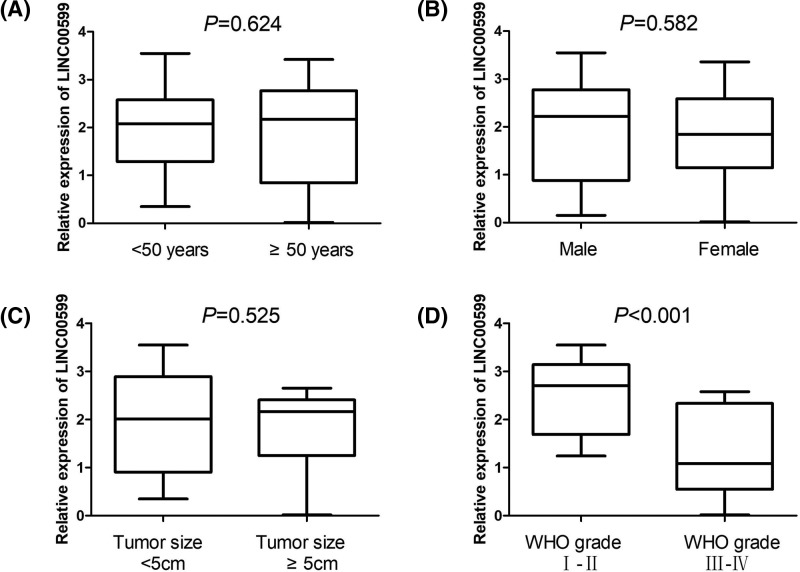
The clinical significance of LINC00599 expression in glioma patients The expression of LINC00599 was estimated in different glioma groups with age (**A**), gender (**B**), tumor size (**C**), and WHO grade (**D**).

For estimating the clinical significance of LINC00599 expression in glioma patients, we grouped 60 glioma tissues based on age (<50 compared with ≥50 years), gender (male compared with female), tumor size (<5 compared with ≥5 cm) and WHO grade (I-II compared with III-IV). We found that glioma tissues with WHO III-IV grade exhibited lower levels of LINC00599 expression than glioma tissues with I-II grade (*P*<0.001, Figure 3D). However, we did not find any difference of LINC00599 expression in groups with age (*P*=0.624, Figure 3A), gender (*P*=0.582, Figure 3B), and tumor size (*P*=0.525, Figure 3C).

**Figure 4:**
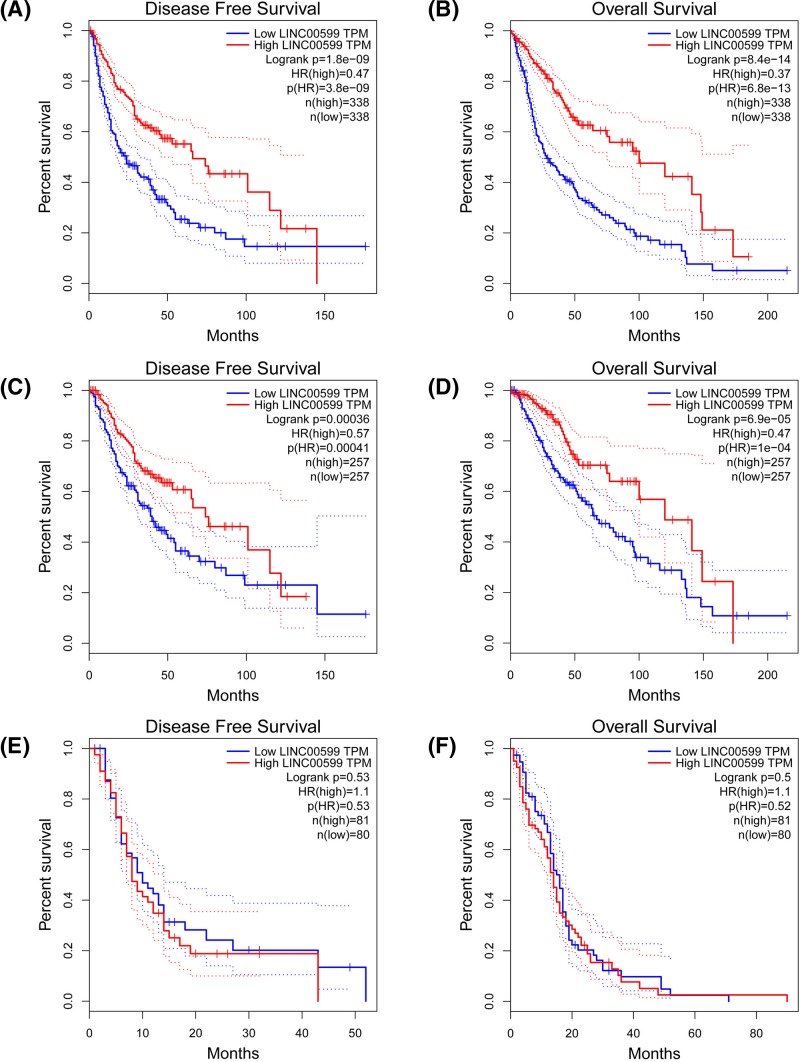
The prognostic significance of LINC00599 expression in glioma patients (**A**) The relationship between LINC00599 expression and disease-free survival in glioma patients. (**B**) The relationship between LINC00599 expression and overall survival in glioma patients. (**C**) The relationship between LINC00599 expression and disease-free survival in LGG patients. (**D**) The relationship between LINC00599 expression and overall survival in LGG patients. (**E**) The relationship between LINC00599 expression and disease-free survival in GBM patients. (**F**) The relationship between LINC00599 expression and overall survival in GBM patients.

For evaluating the prognostic significance of LINC00599 expression in glioma patients, we analyzed the relationship between LINC00599 expression and TCGA glioma cohort including GBM (*n*=161) and LGG (*n*=514). We observed that low LINC00599 expression was associated with poor disease-free survival (*P*<0.001, Figure 4A) and overall survival (*P*<0.001, Figure 4B) in glioma patients. Furthermore, we conducted subgroup analysis on TCGA GBM cohort and TCGA LGG cohort. We also observed that LINC00599 expression was negatively associated with disease-free survival (*P*<0.001, Figure 4C) and overall survival (*P*<0.001, Figure 4D) in TCGA LGG cohort, but had no statistical relationship with disease-free survival (*P*>0.05, Figure 4E) and overall survival (*P*>0.05, Figure 4F) in TCGA GBM cohort.

### LINC00599 expression suppresses glioma cell migration and invasion

**Figure 5:**
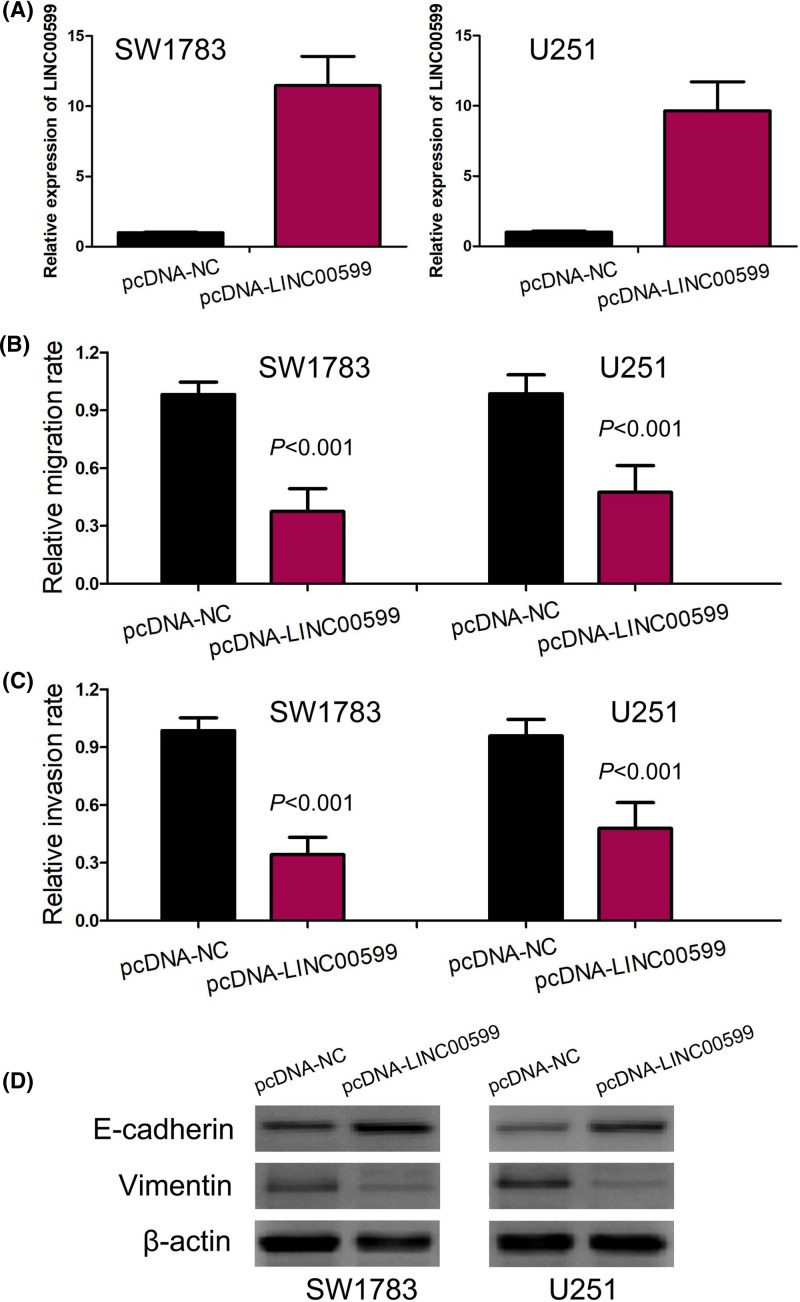
LINC00599 expression suppresses glioma cell migration, invasion, and EMT process (**A**) The pcDNA-LINC00599 caused an obvious up-regulation of LINC00599 in SW1783 and U251 cells. (**B**) Up-regulation of LINC00599 expression depressed glioma cell migration. (**C**) Up-regulation of LINC00599 expression inhibited glioma cell invasion. (**D**) Up-regulation of LINC00599 expression increased E-cadherin expression and decreased vimentin expression in glioma cells. Abbreviation: EMT, epithelial–mesenchymal transition.

Because LINC00599 has been shown to inhibit glioma cell proliferation and promote cell apoptosis [26], we mainly explored the effect of LINC00599 expression on glioma cell migration and invasion though transwell cell migration and invasion assays *in vitro*. The pcDNA-LINC00599 caused an obvious up-regulation of LINC00599 in SW1783 and U251 cells, which was suggested by qRT-PCR (Figure 5A). Then, the results of transwell cell migration and invasion assays suggested up-regulation of LINC00599 expression notably depressed glioma cell migration and invasion (*P*<0.001, Figure 5B,C).

### LINC00599 expression inhibits epithelial–mesenchymal transition process

The epithelial–mesenchymal transition (EMT) process has been confirmed to play a key role in glioma cell migration and invasion [3,4,7]. For detecting the effect of LINC00599 expression on EMT process, the change of epithelial marker (E-cadherin) and mesenchymal marker (vimentin) was measured in glioma cells by Western blot. The result of Western blot indicated that up-regulation of LINC00599 expression remarkably increased E-cadherin expression and decreased vimentin expression, which suggested that LINC00599 inhibited EMT process in glioma cells (Figure 5D).

## Discussion

In the recent decade, LINC00599 has been suggested to be involved in physiological and pathological processes, such as hippocampal axogenesis [15], retinal cone survival [15], atherosclerosis [16], neurodevelopmental disorder [8], retinal reactive gliosis [6], myeloid-derived suppressor cell differentiation [18], and cigarette smoking [10]. Nowadays, growing studies reveal the role of LINC00599 in human tumorigenesis. Morandi et al. [9] performed DNA methylation analyses in oral squamous cell carcinoma tissues, high-grade squamous intraepithelial lesions and corresponding normal contralateral mucosae, and found oral squamous cell carcinoma tissues and high-grade squamous intraepithelial lesions exhibited hypermethylation of LINC00599 in comparison with corresponding normal contralateral mucosae. In addition, Tian et al. [19] detected LINC00599 expression in prostate cancer tissues and cell lines, paired normal prostate tissues and normal prostate epithelial cell line, and observed that levels LINC00599 expression were higher in prostate cancer tissues and cell lines than in paired normal prostate tissues and normal prostate epithelial cell line, respectively. However, LINC00599 expression was suggested to be reduced in glioma. Initially, Reon et al. [14] conducted an *in silico* analysis in glioma and normal brain tissues, and found that LINC00599 expression was markedly decreased in glioma tissues compared with normal brain tissues. Then, Zhang et al. [26] further found that LINC00599 expression levels were reduced in glioblastoma cells lines compared with NHAs. In our study, we provided more evidence about the expression pattern of LINC00599 in glioma. We first observed the expression of LINC00599 in 31 types of human cancers including tumor tissues and corresponding normal tissues at TCGA database, and found that the expression of LINC00599 was only reduced in LGG tissues and GBM tissues compared with normal brain tissues. Then, we performed qRT-PCR to confirm the expression of LINC00599 in glioma tissues and cell lines, and also found LINC00599 expression was decreased in glioma tissues and cell lines compared with matched adjacent normal tissues and NHAs, respectively. In summary, LINC00599 expression is reduced in glioma tissues and cell lines.

The clinical and prognostic values of LINC00599 expression in glioma patients was further explored. We analyzed the expression difference of LINC00599 in glioma cases, which were grouped according to age, gender, tumor size, and WHO grade, and found glioma tissues with WHO III-IV grade exhibited lower levels of LINC00599 expression than glioma tissues with I-II grade. Conversely, Tian et al. found LINC00599 overexpression was associated with large tumor size, high Gleason score, and advanced clinical stage in prostate cancer patients [26]. Meanwhile, they estimated prognostic value of LINC00599 expression in prostate cancer patients, and found patients with low expression of LINC00599 had obviously better overall survival than patients with high expression of LINC00599 [26]. In the study, we further investigated the prognostic value of LINC00599 expression in glioma patient through analyzing TCGA database, and found low LINC00599 expression was associated with poor disease-free survival and overall survival. Furthermore, we conducted subgroup survival analysis according to glioma types, and found that LINC00599 expression was negatively associated with disease-free survival and overall survival in TCGA LGG cohort, but had no statistical relationship with disease-free survival and overall survival in TCGA GBM cohort. The difference between LGG cohort and GBM cohort may be due to the limited sample size in GBM cohort.

LINC00599 has been found to inhibit glioma cell proliferation and promote cell apoptosis through modulating miR-185-5p/KLF16 axis [19]. The effect of LINC00599 on glioma cell migration and invasion were still unclear. In our results, we found up-regulation of LINC00599 depressed glioma cell migration and invasion through regulating EMT process. However, Tian et al. showed LINC00599 as a tumor-promoting lncRNA to regulate cell proliferation, colony formation, and invasion through modulating miR-185-5p in prostate cancer [26].

In conclusion, LINC00599 expression is down-regulated in glioma tissues and cell lines, and associated with WHO grade and prognosis in glioma patients. Up-regulation of LINC00599 depresses glioma cell migration and invasion through regulating EMT process.

## Supporting information

**Supplementary Figure:** 

## Abbreviations

EMT: epithelial–mesenchymal transition
FBS: fetal bovine serum
GBM: glioblastoma multiforme
LGG: lower grade glioma
lncRNA: long non-coding RNA
NHA: normal human astrocyte
qRT-PCR: quantitative real-time PCR
TCGA: The Cancer Genome Atlas
