# Supplementary material for: Low LINC00599 expression is a poor prognostic factor in glioma
Source: Biosci Rep. 2019 Apr 2;39(4):BSR20190232. doi: 10.1042/BSR20190232 (PMC6443953; doi:10.1042/BSR20190232)
Supplement: Supplementary file 1 [file bsr20190232_Supp1.pdf]

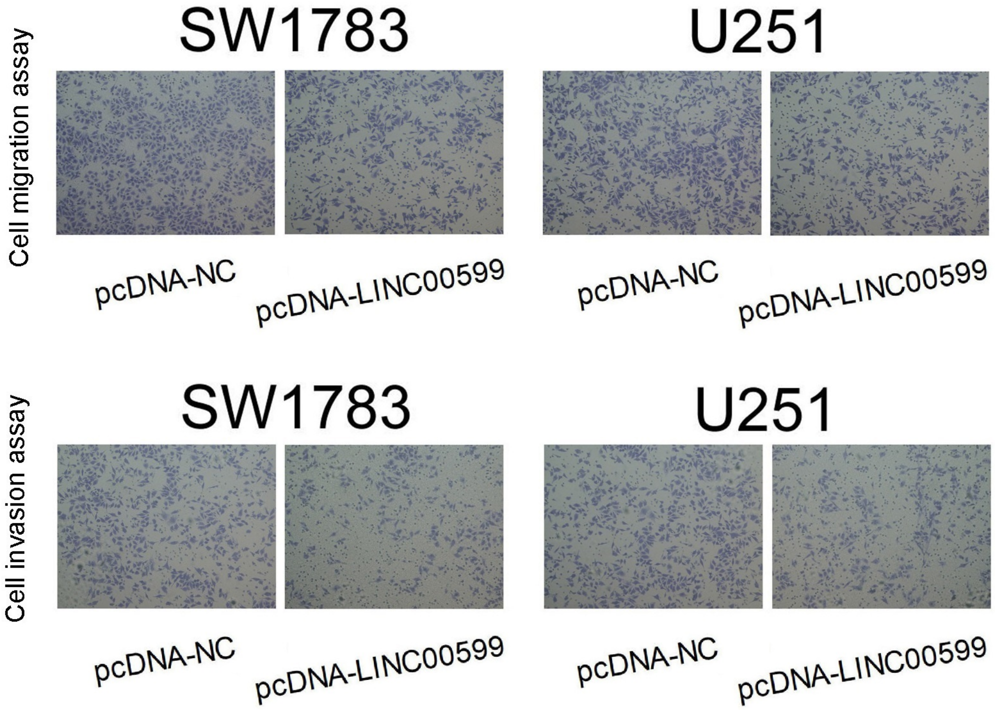

Cell migration assay
SW1783
U251
pcDNA-NC
pcDNA-LINC00599
pcDNA-NC
pcDNA-LINC00599
Cell invasion assay
SW1783
U251
pcDNA-NC
pcDNA-LINC00599
pcDNA-NC
pcDNA-LINC00599

>ENST00000517675.1 LINC00599 cdna:lincRNA
AATGGGGCTGGCTGAGCACCGTGGGTCGGCGAGGGCCCGCCAAGGAAGGAGCGACCGACC
GAGCCAGGCGCCCTCCGCAGACCTCCGCGCAGCGGCCGCGGGCGCGAGGGGAGGGGTCTG
GAGCTCCCTCCGGCTGCCTGTCCCGCACCGGAGCCCGTGGGGTGGGGAGGTGTGCAGCCT
GTGACAGACAGGGGCTTAGAGATGCAAACAGACTCAGGGAGAGAAACAGAAGCTGATTCT
GTGACAGAAGCAGATCTGTGCAGCACAGATGCGGTGTGCGTGGGGAGGGGGTCGCCTGGG
AGCGCATTGCGGAGTGCTTGTGTGTGCAGATTTTTCTCTGGGCTCAGGACTCATTGTATG
TGGGTCAACACCTTCCTCCGTGACTGTGTTTTTGTTCTGAGCTGAGTTTTTTGGTTTGCC
CTTAAAAAAATAATAATTTGGCATCCAGAGACTGGCAGACTGCCTCAGGGCCTGGACTGC
GGATATATTGTGTTCTGCTTGAGGTTTGGGGAGGAGGGCAGGCGGTAGGAAGGGAGAGGG
GGAGCTGTTTGTCACACTTTGCTGTAGAGCTGAGAGCACCTGACAAGCTTAAGGAAGTCG
TTGGGCTATGTGGACAAGAAGGAGCCAGCTCCCAGCGGGTTCACAAGCTCTATCGGAGTT
GAAAGCGTGGTCATGGCTCTAAGGAGCACCTCACGCCCTCCCTGTAGCTGTTATTGCAGT
TTCAGGCAGAGATCCAGGAGCTGCAGAGGAAGGGAGAGGCACAATAACCTACATGGACCC
AAGGGAGACATGTGTTCCTTTAAAAATGTGAACAGAAGGAAAAACAGAATGTGTGCAACT
GGGGGTCTGAGGAAAGACTGTTTTGAAAGAGGCTGTCAGGGAATGGAAAGGGTTAAGCTT
TTCATCCTGAAGAACCTGCTTCCCAAATCAGGCCTTCCTCCCATCACTAGACCCTGAGCA
GCAGCTGGTCCTAGAGACCCCCCTCGTACTGCGCTGCCACAGTCTCATCCCATTTCCAGC
TCTGTATTAACCACCAAGCTGCAGCGGATGGGGCAAGACCTAAGCTCATTAATTTCCAGG
TGGAGGAAATGGAGGAGGGGCAGGTCCCTGCAGTAGAGGGAGGAGCAGAGAAAGGAGGCC
AAGAGCTGGATCCTTCTGCCCAGGAAGCCTGCTGCATCCCTTCCCCCGAGCATGGCAGAG
GCCTGGCTTTGCAAGGCCAAGGCCATAAGGGATGCTTAGGAGATTAATTTGATTCCTGAC
ACAATAATCAAGCCCTAAGAGTCTCCACTGAAGCTTACTGAGGACTTCTTTCCTCTCCAA
AGCCTCAGTCTAGCCTGCTAAATAAATTAGTATTCAGTGATGCCTTGGATCAGGGCCCCT
CCCCGGCCTCAGTTTCCCCAAATATTTATTAAGTACCTACTGTGTGCAATCCTTGTGTAA
TTATTAACTCTTAGGCTCTTCATTTGCCCTCCTAAAGCAGTGTTTAGAGTCAGGCAGAGG
TTAAGTGTGTTGCACCTCACCTAGATACTTCCAGAACCTTCTCTGGGTCTGCAGAATGTG
GCACAACCTGCTTGCCCCCGCAGAGAGAAAGCTGCAGTGCACATCCTGCAGACTGCAGGT
GCTGGGCTGCCTCTGGAGTCCCAGAAGGCAAGCTTGGCTGCAGGACAGAAAGGGAGAACA
GCTTCTCTCACCCCTGAGCCTTCACAAGCCCTTGTCTATTTGCCGTTGCCTTCAAAATAT
ACCTCCCCCGAAACCAGTAGCTTTCTGAGTCCTGGTGTCCCCTCCGCCCTTTCTGGACAG
GTTTGGGAAGAAGAAAGCAGTCAGTGCTGGGCCTTATTGGGGTGTGAAGCGCCTTGCTCT
GCCCCTTCTGCTCACTGTGAAGGCCGCTGGATGCTTCTCTTAGGCATGGTTTAAGCCTCC
GATTACTAAACCCCTTGCCCCACAAACGTCCACATTGACGAGCCTCTTTTTAGTAACTGC
TTCCCCGTAATTCCTTCAGAGGTTGCTGTACCCTTCGCTGATGTGCTGCCCTCCTGTAAA
ACCTCCAGATGCCTTCCCACGTAATGCCCCTTTCAGATGCTTTAAGCTGAGAGCTTAAAC
CACAGGTACCATGGCTGACGCCTGCCAGGTTTCTGCTGCAGATAATCTATGATGGGAGGG
GCATATTTTTTACTTCATTACTTATGTAAACTCTTGTTCCAGAAAGCTTTAATGTGTGTG
GGAGTGTTCTGGGTCTATTAGGTCTGTGCGCATGGGTGTGGGCATTTGCCTGTGTCCACC
GGGTGGGTCTCATTATGAAATGTATGTTTATGTAGGGCTTTAATGGCTGAAAATGGCAAA
GAGATGAATAGACCACTTGGCCCCATGTGTAATTGCCAGGCCCCTTCTGTGCTCAAATGA
GGTGTCCGAGTGAAGGTCAGCCCTTCCCTTCTGTATTTGGGGCCTATTTATGCCACCAGT
AATTTTATAAGAAATCTGAATAGTTCTCCCCTTTGAGTGCATTTAACTCTTTAGTATCTT
CTCTCTTACCTATTTGAGCCCCTCTAGCTACAGTCTGGCTTAAATGAAAGGGGAATTATA

TGCTTAAGAAAAAGTAGGACACGGTTGAGGCAGTTTGCTGACTGAATACGCGAAGAAGGA
CCTGATGGGCTCATATGCACCACTGCCATCACAGTCCCCATCGTGATGCAAGCTTATATG
ATTCTTGAGGTAACTCTACCAGATACTTCCAGATTTAGAAATGTGTCAAAGGAAAAATTG
GTGATACTCTTCTTTCCCCTGCCAGAAACAGCCCAGATCTCCTCTTAAGCGGAAAAGAGA
TTGACCTTCTAGCAGAGGCAAAGGTAAACTCCTGTAAGTTACTTCTGTTACCAAAGGGAG
GGGGGCGGCTTTTGTGAATGTATGAGGAGCTTTTGCCAGAGAGATATTCGGAGGAGGGGT
GTGCCCATATGCACACATATATTTTCCCGCATAACCGTATCCAATGCTAGCATTTAGAGG
AAGGCATTTAGCCACCAAAAGTCCATCCATCTATGCTGCTTCCACAGAGAAAACATTTTC
TCTTTCCTCCTCTTGAACTTACATAATATCCTCCTCCCATTCCAACCTTAGAATGGAGTC
TTCTGGGGGCAGCTGCAAAGCGTTCTCCCTAGGACAGATGGAGCCTCCCTTTCCTCATCT
ACTCTGTGGGTGGTTTCAGGGCCCACGAGTCAACATGAGGAGTTGTGCTGGTGGTATGTG
TGTTGGAGGCTGGGCTGGCTGATTCACAGTGACGAGGATGTCAATAATAACAAGAATGAG
AATGATGGTACCTAATAAAGACTTTTTTTCCCAA
>LINC00599 cds:lincRNA
